# Qiwei granules alleviates podocyte lesion in kidney of diabetic KK-Ay mice

**DOI:** 10.1186/s12906-015-0603-x

**Published:** 2015-03-31

**Authors:** Jingxin Zhou, Wen Sun, Hisae Yoshitomi, Linyi Li, Lingling Qin, Xiangyu Guo, Lili Wu, Yan Zhang, Xinli Wu, Tunhai Xu, Ming Gao, Tonghua Liu

**Affiliations:** Beijing University of Chinese Medicine, 11 North 3rd-ring East Road, 100029 Chaoyang District, Beijing, People’s Republic of China; Dongzhimen Hospital Eastern Affilated to Beijing University of Chinese Medicine, 116 Cuiping West Road, 100029 Tongzhou District, Beijing, People’s Republic of China; School of Pharmaceutical Sciences, Mukogawa Women’s University, 11-68 Koshien Kyuban-cho, Nishinomiya, Hyogo 663-8179 Japan

**Keywords:** Qiwei granules, Diabetic nephropathy, KK-A^y^ mice, Podocyte, Slit diaphragm, Akt signaling pathway

## Abstract

**Background:**

Chinese medicine comprised of all natural herbs is widespread used in the treatment of diabetic nephropathy (DN). Podocyte contributes to the integrity of glomerular filtration barrier whose injury plays an important role in the initiation and progression of DN. Our study aimed to investigate the effect of Qiwei granules on podocyte lesion in diabetic KK-A^y^ mice kidney and its underlying mechanism.

**Methods:**

Twelve-week-old male KK-A^y^ mice were randomly divided in vehicle group and Qiwei granules group, while C57BL/6J mice were used as normal control. The mice were gavage with 1.37 g/kg/day Qiwei granules or water for 10 weeks. We measured water, food intake and body weight (BW) and fasting blood glucose (FBG) every 2 weeks, and urine protein every 4 weeks. At the end of the experiment, all surviving mice were sacrificed. The kidney weight and serum renal parameters were measured, and the renal morphology was observed. To search the underlying mechanism, we examined the podocyte positive marker, slit diaphragm protein expression and some involved cell signal pathway.

**Results:**

Qiwei granules treatment significantly improved the metabolic parameters, alleviated the urinary protein, and protected renal function in KK-A^y^ mice. In addition, the glomerular injuries and podocyte lesions were mitigated with Qiwei granules treatment. Furthermore, Qiwei granules increased expression of nephrin, CD2AP, and integrin alpha3beta1 in the podocytes of KK-A^y^ mice. Qiwei granules improved the phosphoration of Akt and inhibited cleaved caspase-3 protein expression.

**Conclusion:**

These finding suggest that Qiwei granules protects the podocyte from the development of DN via improving slit diaphragm (SD) molecules expression and likely activating Akt signaling pathway in KK-A^y^ mice.

## Background

Diabetic nephropathy (DN), one of most common and serious complication of diabetes, is a major cause of end-stage renal disease (ESRD) [1]. Diabetic nephropathy is characterized with expansion of cellar and matrix components in the mesangium, thickness of glomerular basement membrane leading to glomerulosclerosis followed by persistent albuminuria and progressive renal dyfunction [2]. However, much attention has been focused on the importance of podocyte injury in the initiation and progression of DN [3,4]. Podocytes are terminally differentiated and highly specialized cells with foot processes attaching on glomerular basement membrane (GBM) and interconnected by slit diaphragm (SD). Podocytes have function to establish the filtration barrier, and remodel the GBM [5]. Nephrin, one transmembrane protein component of SD, is essential to maintain normal podocyte structure and control the filtration barrier [6]. CD2AP, another transmembrane protein locates at podocyte slit membrane interacting with nephrin to maintain the structure of podocyte [7]. A number of studies suggest that both the protein and mRNA expression of nephrin were significantly decreased in diabetic nephropathy patients and animals [8,9]. However, some evidence indicates that SD maintains podocyte functional integrity not only because of foundation for the filter barrier but also participation in cell signaling such as PI3K/Akt [10]. Intergrins are transmembrane molecules assembled of α and β subunits adhered on GBM to regulate the shape and adhesion of the podocytes [11]. The integrin α3β1 in the podocyte foot processes interacted podocyte-GBM mediates cell adhesion and is associated with integrin signaling in charge of glomerular filtration barrier. It has been reported that expression of integrin α3β1 on podocytes are reduced in diabetes patients and rats [12]. There are no podocyte foot processes in the mice born lack of integrin α3 [13].

Chinese medicine comprised of all natural products is widespread used in the treatment of diabetes and its complications, which have been proved to alleviate the progression of diabetic nephropathy [14]. Qiwei granules (used name Zhi xiao tong mai ning) is composed of Astragalus membranaceus, Rehmannia glutinosa, Prunella vugaris, Curcuma zedoaria, Euonymus alatus, Panax pseudoginseng, and Rheum officinale.

It has been reported that Astragalus membranaceus, and Euonymus alatus both protects diabetic nephropathy kidney in animals [15,16], Rehmannia glutinosa decreases the glucose in diabetic rats [17] and Prunella vulgaris [18] improves diabetes and its complications in vivo and vitro [19,20]. Many studies have been suggested that Panax notoginseng improves glucose and lipid metabolism, prevents oxidation, and ameliorates urinary albumin in patients with chronic renal failure [21,22]. Previous clinical observation has shown that Qiwei granules decreased blood glucose and reduced the proteinuria to prevent diabetic nephropathy [23]. It also has been demonstrated that Qiwei granules prevented the progression of tubulointerstitial fibrosis in KK-A^y^ diabetes mice by TGF-β1/Smads signaling [24]. But the underlying mechanism on podocyte was not unclear.

KK-A^y^ mouse was chosen as a diabetic nephropathy model in our study. KK-A^y^ mouse is established by transfection of the yellow obese gene (A^y^) into moderate KK mouse [25]. KK-A^y^ mouse is spontaneously developed and characterized of T2 Diabetes such as obesity, hyperglycemia, insulin resistance which is widely used as a T2D model [26]. Furthermore, KK-A^y^ mouse over 12 weeks age appears albuminuria, and diffuse mesangial matrix expansion, segmental glomerulosclerosis [27]. Therefore, KK-A^y^ mice are considered as good diabetic nephropathy models renal lesions of which are similar with those of human.

In the present study, we investigate whether Qiwei granules protect the podocyte and the underlying mechanism in the KK-A^y^ diabetic mice kidneys.

## Methods

### Qiwei granules

Qiwei granules were prepared by Shanxi JinYu Pharmaceutical Company., Ltd. Qiwei granules consists of Astragalus membranaceus, Rehmannia glutinosa, Prunella vulgaris, Curcuma zedoaria, Euonymus alatus, Panax pseudoginseng, and Rheum officinale.

### Reagents

The antibodies against integrin beta1 (integrin β1), pAkt (Ser473), Akt, cleaved caspase-3 and caspase-3 were purchased from Cell Signaling Technology, Inc. (Beverly, USA). Nephrin and integrin alpha3 (integrin α3) antibodies were purchased from R&D Systems, Inc. (MN, USA). CD2 associated protein antibody (CD2AP) was purchased from Abcam, Inc. (MA, USA). In-situ cell death detection kit POD was purchased from Roche Diagnositcs (Mannheim, Germany). Wilms tumor −1 antibody (WT-1) was purchased from Santa Cruz Biotechnology, Inc. (Texas, USA).

### Animals

Eight-week-old male KK-A^y^ mice and C57BL/6J mice were purchased from institute of Laboratory Animal Science of Chinese Academy of Medical Sciences (Beijing, China). All mice were housed in same controlled conditions under temperature of 25 ± 1°C and humidity of 55% ± 5% on a regular 12 h light/dark cycle with free access to food and water. The KK-A^y^ mice were fed with high-fat diets (458 kcal/100 g, containing 10% fat), and C57BL/6J mice were fed with normal diets. After acclimating to new surroundings for 4 weeks, KK-A^y^ mice were randomly divided into two groups of 6 mice each, Qiwei granules group and vehicle group, while C57BL/6J mice (n = 6) were used as normal control group. The mice were gavage with 1.37 g/kg/day Qiwei granules (Qiwei granules group) or same volume water (vehicle group and normal control group) as administration. Water, food intake and body weight (BW) of mice and fasting blood glucose (FBG) levels were measured every 2 weeks. All mice were placed into metabolic cages for urine collection at 0, 4, 8 weeks of treatment. After 10 weeks of treatment, all surviving mice were fasted for 12 h and sacrificed. The kidney were removed, cleaned for measurement of right kidney weight, and promptly frozen in liquid nitrogen. And portions were appropriated in 4% paraformaldehyde for histological analysis and in 2.5% glutaraldehyde solution for electron microscopic examination.

The study was approved by the Ethical Committee of Beijing University of Chinese Medicine, which was carried out in accordance with the Principles of Laboratory Animal Care published by National Institutes of Health.

### Blood and proteinuria analysis

The blood was collected and sera were prepared by centrifugation, and measured by Beckman coulter. Serum urine nitrogen (BUN) and serum creatinine (Scr) were measured using the corresponding commercial kit (Leadman, Beijing). 24 h urinary samples were collected from mice in metabolic cages without restriction of water intake at the 0, 4, 8 weeks of treatment. After collection, urine volume was recorded and determined of 24 h urinary protein by Bradford method.

### Histological analysis

Parts of kidney tissue samples were fixed in 4% paraformaldehyde and embedded in paraffin. Under light microscopy, 2-3 μm sections were prepared and stained with hematoxylin and eosin (HE), Masson’s Trichrome, and periodic acid-Schiff (PAS). For PAS stain, the mesangial matrix expansion was evaluated in 10 randomly selected glomeruli in the cortex per animal under high magnification (×400) with Image-Pro Plus 6.0 (Media Cyemetics, Siliver Spring, MD). The area of fibrosis was investigated by measuring the collagen deposition staining by Masson’s Trichrome under × 200 magnification using Image-Pro Plus 6.0 software. 1 mm^3^ samples of renal cortex were also fixed in 2.5% glutaraldehyde solution, rinsed in phosphate buffer, fixed in osmic acid and embedded in epon. Semithin 1 μm sections were cut and stained with uranyl acetate and then lead in nitrate solution. Representative glomeruli from 5 mice, each from untreated KK-A^y^ mice, KK-A^y^ mice treated with Qiwei granules, and normal control mice were examined. Images were acquired on a JEOL JEM-2100 transmission electron microscope (JEOL, Tokyo, Japan).

### Immunohistochemical staining for TUNEL and WT-1

TUNEL was performed with the In-situ cell death detection kit POD to detect apoptotic positive cells. Staining for podocytes in renal tissues was performed using the protocol of polyclonal rabbit anti-mouse WT-1 antibody (1:200). Negative control for immunohistochemistry was run incubating with nonimmune serum instead of the primary antibody. For quantitative determination of podocyte numbers, the WT-1 positive cells were calculated in a total of 40 glomeruli area by Image J software. The averages of podocyte numbers per glomerulus were assessed from individual five mice each group.

### Western blotting analysis

The kidney tissue was homogenized with ice-cold homogenized buffer containing 50 mM Tris–HCl (8.0), 150 mM NaCl, 0.025% NaN3, 0.1% SDS, 0.1 mg/ml PMSF, 0.01 mg/ml Aprotintin, 0.5% Na deoxycholate, and 0.1%Nonidet-P40. After incubation on ice for 20 min, lysates were centrifuged at 10000 rpm for 10 min and supernatants were isolated. Proteins were extracted by boiling in 0.5 mmol/ml Tris–HCl, PH 6.8, 10% SDS, 20% glycerol, 0.05% bromophenol blue, and 2-mercaptethanol. The proteins (40 μg/sample) were electrophoresed on a 7.5%-12.5% sodium dodecyl sulfatepolyacrylamide gel electrophoresis (SDS-PAGE) at 100 V for 2 h and transferred onto polyvinylidene fluoride (PVDF) membranes (Merck Millipore, Germany) at 30 mA for 2 h. The membrane was blocked in 5% skim milk in Tris-buffered saline containing 0.1% Tween 20 for 1 h, and incubated with the primary antibody nephrin (1:1000), CD2AP (1:2000), integrin α3 (1:1000), integrin β1 (1:500), pAkt (1:1000), Akt (1:1000), cleaved caspase-3 (1:1000) and caspase-3 (1:1000) overnight. After washing with TBST and incubated for 1 h with horseradish peroxidase-linked anti-rabbit and anti-mouse secondary antibodies (ZSGB-Bio, Inc., Beijing, China). Detection was achieved using ECL kit (Santa Cruz, USA). GAPDH was used as an internal control. The density of the bands was measured using IPP.

### Quantitative real time RT-PCR

Total RNA was extracted from the kidney using Trizol kit (Invitrogen, USA). Then RNA (1 μg) from each sample was reverse-transcribed to cDNA using the M-MLV RT Kit, according to the manufacturer’s instructions (Takara). THUNDERBIRD SYBR qPCR Mix was used for quantitative real-time RT-PCR analysis of each gene’s expression. The primers are listed as follow: nephrin forward, 5′- TCTGGCGGAGAAGACTGAG -3′; nephrin reverse, 5′- GTGCTAACCGTGGAGCTTCT -3′; integrin α3 forward, 5′- CTGGAGTGGCCCTATGAAGT -3′; integrin α3 reverse, 5′- TGACTCCAGGGTCAGAGAGA -3′; integrin β1 forward, 5′- CTTGCTGCTGATTTGGAAAC -3′; integrin β1 reverse, 5′- CTTCGGATTGACCACAGTTG -3′; GAPDH forward, 5′- GCAAGTTCAACGGCACAG -3′; GAPDH reverse, 5′- CGCCAGTAGACTCCACGAC -3′. Amplification was performed with a real-time PCR system (ABI Prism 7500). The amplification was performed as follows: 94°C for 15 min followed by 50 cycles of 94°C for 15 s, 60°C for 60 s, and 72°C for 10 min with a real-time PCR system (ABI Prism 7500). The data are expresses as a relative value after normalization to the GAPDH expression.

### Statistical analysis

Data are expressed as mean ± SEM and statistically analyzed by Dunnett test. Differences were considered significant for P < 0.05.

## Results

### Effect of Qiwei granules on metabolic and renal parameters

**Figure 1:**
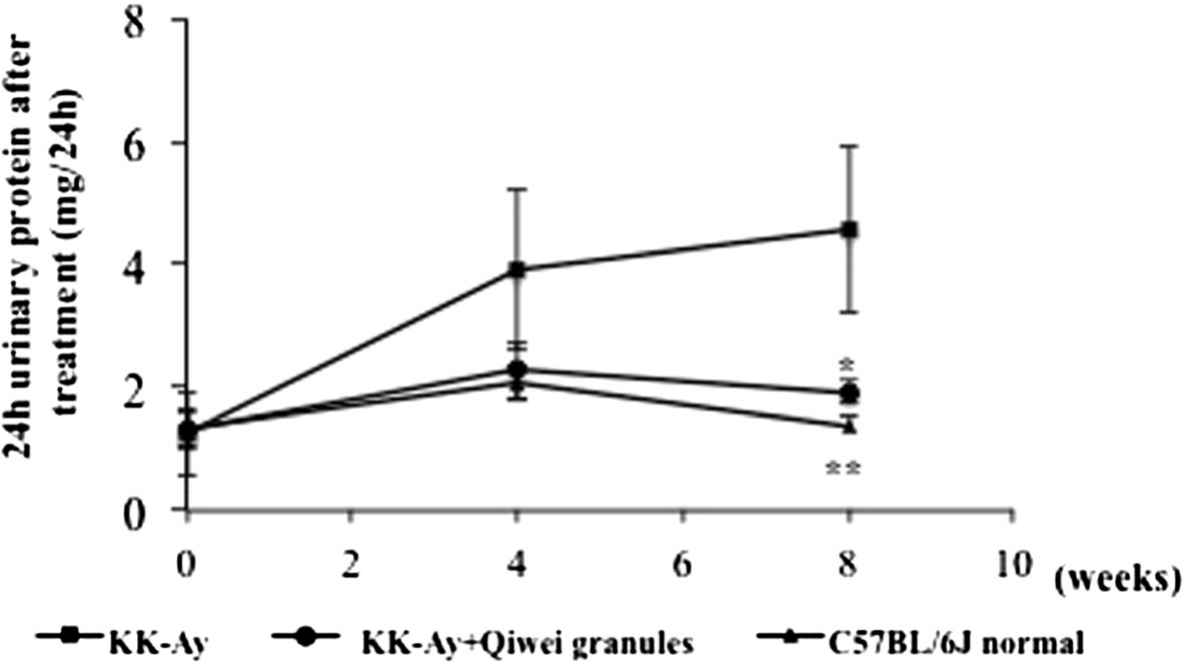
**24 h urinary protein after 0, 4, 8 weeks treatment were measured in mice.** Data of the urinary protein are expressed as mean ± SEM (n = 6). *P < 0.05, **P < 0.01 compared with KK-A^y^ vehicle group.

We measured body weight, fasting blood glucose (FBG) at 12 weeks of age, and confirmed no significant differences between vehicle KK-A^y^ mice and Qiwei granules treated KK-A^y^ mice (Table 1). After 10 weeks treatment, body weight of vehicle KK-A^y^ mice remained a high level compared with normal mice, but did not differ from KK-A^y^ mice with Qiwei granules treatment. At the end of 10 weeks treatment, FBG in vehicle KK-A^y^ mice was higher than normal mice, and Qiwei granules significantly decreased the level of FBG in KK-A^y^ mice (Table 1). The kidney weight levels in Qiwei granules mice tended to be lower than those in vehicle KK-A^y^ mice, although the change was not statistically significant. Moreover, Qiwei granules treatment alleviated the levels of Scr of KK-A^y^ mice close to normal mice compared with vehicle KK-A^y^ mice. 24 h urinary protein in vehicle KK-A^y^ mice was remarkablely increased, and Qiwei granules significantly decreased 24 h urinary protein after 8 weeks treatment (Figure 1). In a word, administration of Qiwei granules treatment showed a positive effect on the FBS, Scr, and 24 h urinary albumin in KK-A^y^ mice.

**Table 1:** **Biochemical parameters in each mouse at 12 and 22 weeks of age**

	**KK-A** ^**y**^	**KK-A** ^**y**^ **+ Qiwei granules**	**C57BL/6J normal**
	**(n = 6)**	**(n = 5)**	**(n = 6)**
**Body weight Initial (g)**	39.67 ± 2.66	39.6 ± 3.49	29.33 ± 1.70**
**Body weight Final (g)**	47 ± 3.79	44.2 ± 5.31	31.33 ± 0.81**
**Initial FBG (mmol/L)**	24.69 ± 4.8	22.28 ± 6.28	5.48 ± 0.79**
**Final FBG** **(mmol/L)**	16.75 ± 9.51	5.8 ± 3.26*	3.98 ± 0.63**
**Kidney weight (g)**	0.30 ± 0.04	0.26 ± 0.05	0.19 ± 0.02**
**Scr (μmol/L)**	44.46 ± 12.17	25.73 ± 7.96**	24.73 ± 6.88**
**BUN (mmol/L)**	6.25 ± 0.73	6.37 ± 1.38	7.75 ± 0.85

Date expressed as means ± SD, *P < 0.05 and **P < 0.01compared with KK-A^y^ vehicle.

### Effects of Qiwei granules on KK-A^y^ mice renal morphology

**Figure 2:**
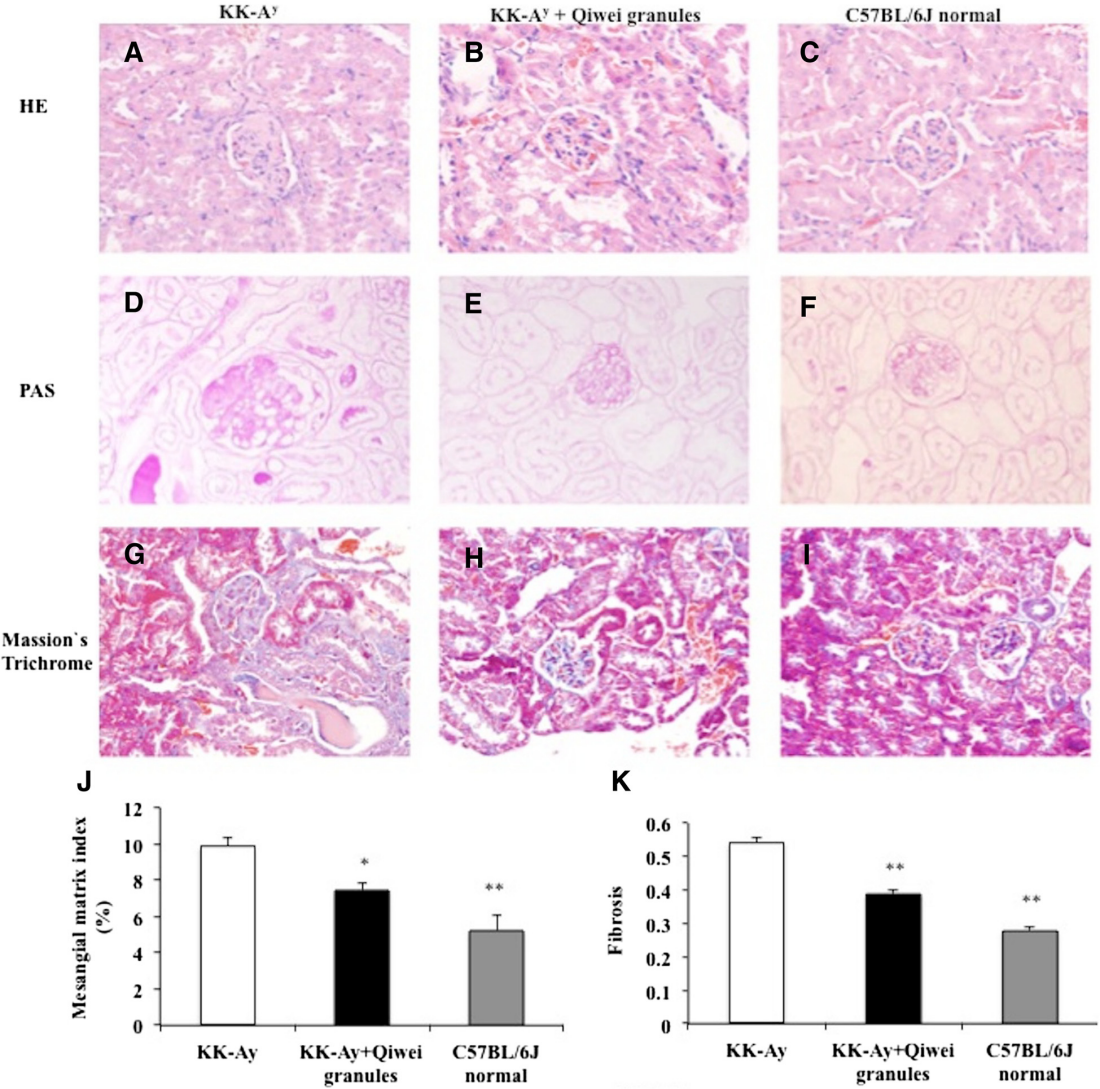
**Renal histopathology was observed after 10 weeks treatment in mice under light microscopy. (A-C)** HE stained glomeruli, ×400 magnification. **(D-F)** PAS stained renal slices, ×400 magnification. **(G-I)** Masson’s Trichrome stained renal sections, ×400 magnification. **(A, D, G)** KK-A^y^ vehicle mice. **(B, E, H)**, KK-A^y^ mice treated with Qiwei granules. **(C**, **F, I)** C57BL/6J normal mice. **(J)** Glomerular mesangial matrix expansion and **(K)** fibrosis of three groups were determined semi-quantitatively by PAS and Masson’s Trichrome staining. Data were presented as means ± SEM. *P < 0.05; **P < 0.01 compared to KK-A^y^ vehicle group.

**Figure 3:**
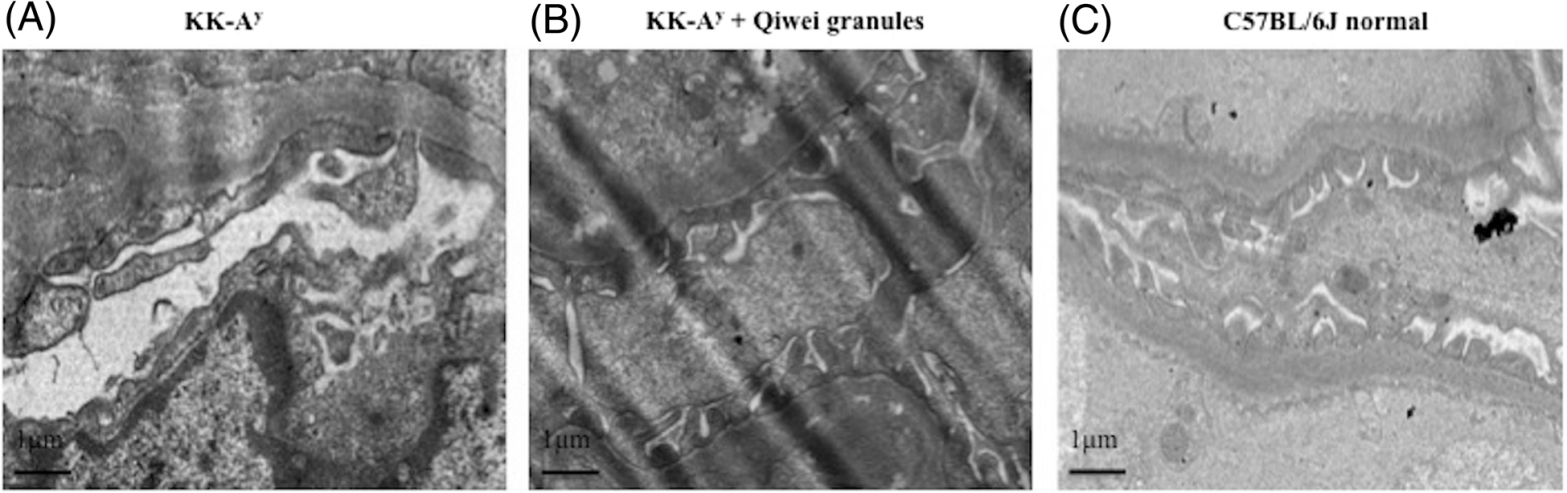
**Ultrastructural features of renal tissue were observed after 10 weeks treatment in three group mice under transmission electron microscopy (bar 1 μm). (A)** KK-A^y^ vehicle mice, **(B)** KK-A^y^ mice treated with Qiwei granules, **(C)** C57BL/6J normal mice.

The renal structure of each group was observed under light microscopy and transmission electron microscopy. Under light microscope, the glomerular changes among three group mice at 22 weeks of age were shown in Figure 2. The predominant observations of vehicle KK-A^y^ mice glomeruli were diffuse mesangial matrix expansion and capillary wall thickening (Figure 2A, G), which were considered a hallmark of diabetic nephropathy. Moreover, this mesangial matrix increase produces the formation of Kimmelstiel-Wilson Nodules in diabetic renal glomerular tuft (Figure 2D). In Figure 2A, segmental sclerosis was present in some KK-A^y^ mice glomeruli compared with normal mice. However, mesangial matrix expansion and capillary thickness in KK-A^y^ mice were reduced by Qiwei granules treatment (Figure 2B, E, H). The reduction of mesangial matrix expansion and fibrosis significantly differed with vehicle KK-A^y^ mice (Figure 2J, K). To investigate whether Qiwei granules affected the renal ultra microstructure, glomerular podocytes were examined by transmission electron microscopy. In Figure 3, the foot processes of podocyte were fusion and the GBM became thicker in KK-A^y^ vehicle mice compared with normal mice. With the treatment of Qiwei granules, the podocyte injury especially the foot process fusion was ameliorated. These results showed that Qiwei granules mitigated the renal injury of KK-A^y^ mice both under light microscopy and transmission electron microscopy.

### Effect of Qiwei granules on apoptosis and podocyte marker WT-1 in KK-A^y^ mice kidneys

**Figure 4:**
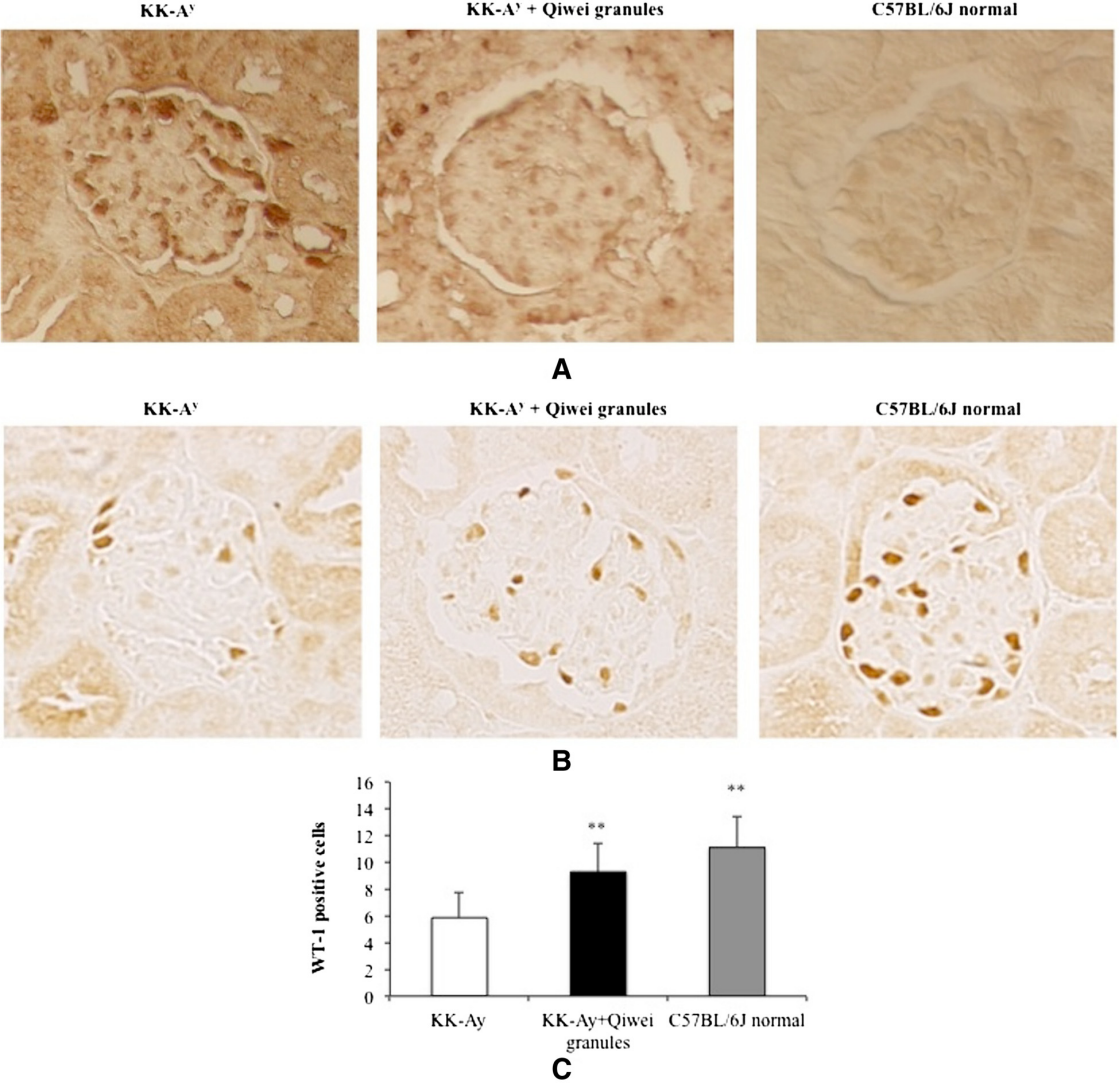
**Photomicrograph of cell apoptosis and podocyte marker WT-1 in KK-A** ^**y**^ **mice kidney. (A)** The apoptotic cells were labeled with TUNEL (×400) in mice kidney. **(B)** The podocytes were perfumed by immunohistochemistry staining of the renal tissues for WT-1 (×400). **(C)** The average WT-1 positive podocytes per glomerulus were expressed as means ± SEM (n = 40). **P < 0.01 compared to KK-A^y^ vehicle group.

Many podocytes, mesangial cells, and endothelial cells were positively labeled by TUNEL in mice kidney. The level of TUNEL positive cells in KK-A^y^ mice was higher than normal mice, while Qiwei granules prevented the cell apoptosis compared with KK-A^y^ mice in Figure 4A. WT-1 is a positive marker of podocyte in kidney. As shown in Figure 4B, WT-1 positive cells were significantly decreased in the glomeruli of KK-A^y^ mice compared with normal mice. However, Qiwei granules preserved WT-1 positive cells in KK-A^y^ mice. The average number of podocytes stained with WT-1 antibody per glomerulus in Qiwei granules treatment group was higher than that in the vehicle group (Figure 4C). These results indicated that Qiwei granules prevented apoptosis and preserved the podocyte in KK-A^y^ mice.

### Enhancement of Qiwei granules on expression levels of nephrin and CD2AP in KK-A^y^ mice renal tissue

**Figure 5:**
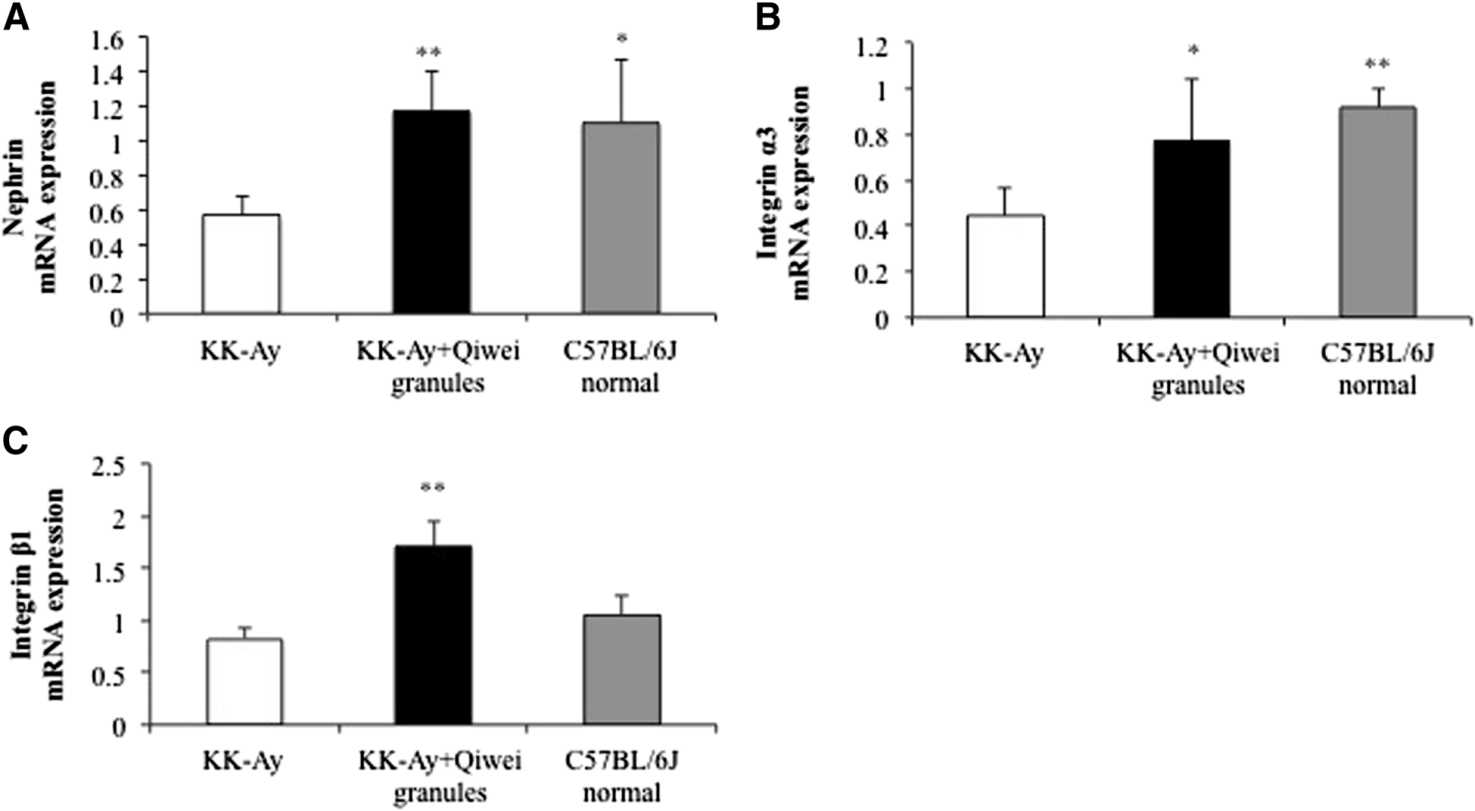
**The mRNA expression levels of slit diaphragm molecules in KK-A** ^**y**^ **mice renal tissue.** The mRNA expression levels of **(A)** nephrin, **(B)** integrin α3, and **(C)** integrin β1 were assessed by real-time PCR. The data were expressed as means ± SEM (n = 5) normalized to GAPDH mRNA expression. *P < 0.05; **P < 0.01 compared to KK-A^y^ vehicle group.

**Figure 6:**
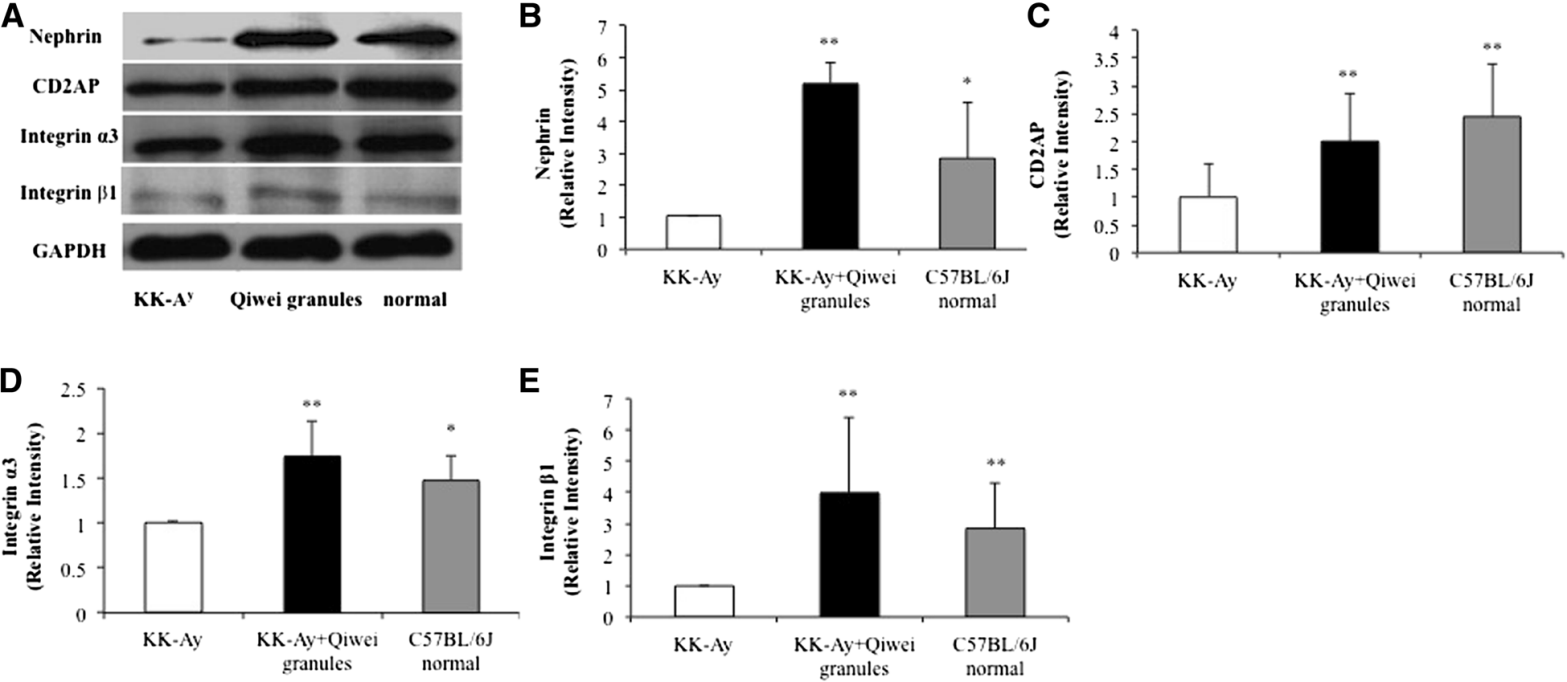
**The protein expression levels of slit diaphragm molecules in the KK-A** ^**y**^ **mice kidney. (A)** Protein expression levels of nephrin, CD2AP, integrin α3, and integrin β1 were analyzed by western blotting in renal tissues of three groups. **(B-E)** Data were presented as mean ± SEM (n = 5) normalized to GAPDH protein expression. *P < 0.05; **P < 0.01 compared to KK-A^y^ vehicle group.

Nephrin is a major transmembrane protein in the podocyte slit diaphragm which relates to renal tissue damage in DN [9]. As shown in Figure 5A, we found that nephrin mRNA expression in the renal tissue of KK-A^y^ mice was lower than normal mice. And the level of nephrin mRNA in Qiwei granules treated KK-A^y^ mice was significantly higher than vehicle KK-A^y^ mice. We further examined the expression of nephrin protein in three groups. As expected, the western blot analysis demonstrated that nephrin protein expression was reduced in vehicle treated diabetic mice (Figure 6A, B). Qiwei granules markedly alleviated the reduction of nephrin protein expression in diabetic kidney (Figure 6A, B). CD2AP, another transmembrane protein, plays a great role in maintenance of structure and function of podocyte. The protein expression of CD2AP was decreased in vehicle KK-A^y^ mice, and treatment with Qiwei granules ameliorated the decrease (Figure 6A, C). These data suggested Qiwei granules improved expression of nephrin and CD2AP protein expression in KK-A^y^ mice renal podocytes.

### Regulation of Qiwei granules on expression of integrin α_3_ and integrin β_1_ in KK-A^y^ mice renal tissue

Integrin α3β1 mediates the adhesion of podocytes to the glomerular basement membrance (GBM) which contain a transmembrane region and intracellular region. So we investigated both the mRNA and protein expression of integrin α3β1 in KK-A^y^ mice kidney. As reports shown, there were significantly downregulation of integrin α3 and integrin β1 mRNA expression in vehicle KK-A^y^ mice compared with normal mice (Figure 5C, D). In addition, the protein expression of integrin α3 and integrin β1 also decreased in vehicle KK-A^y^ mice (Figure 6A, C, D). However, the decreases in mRNA and protein expression of integrin α3 and integrin β1were obviously ameliorated with Qiwei granules treatment in KK-A^y^ mice. These results suggested that Qiwei granules regulated the protein and mRNA expression of integrin α3 and integrin β1 in KK-A^y^ mice.

### Effect of Qiwei granules on pAkt /Akt and cleaved caspase-3/caspase-3 in KK-A^y^ mice renal tissue

**Figure 7:**
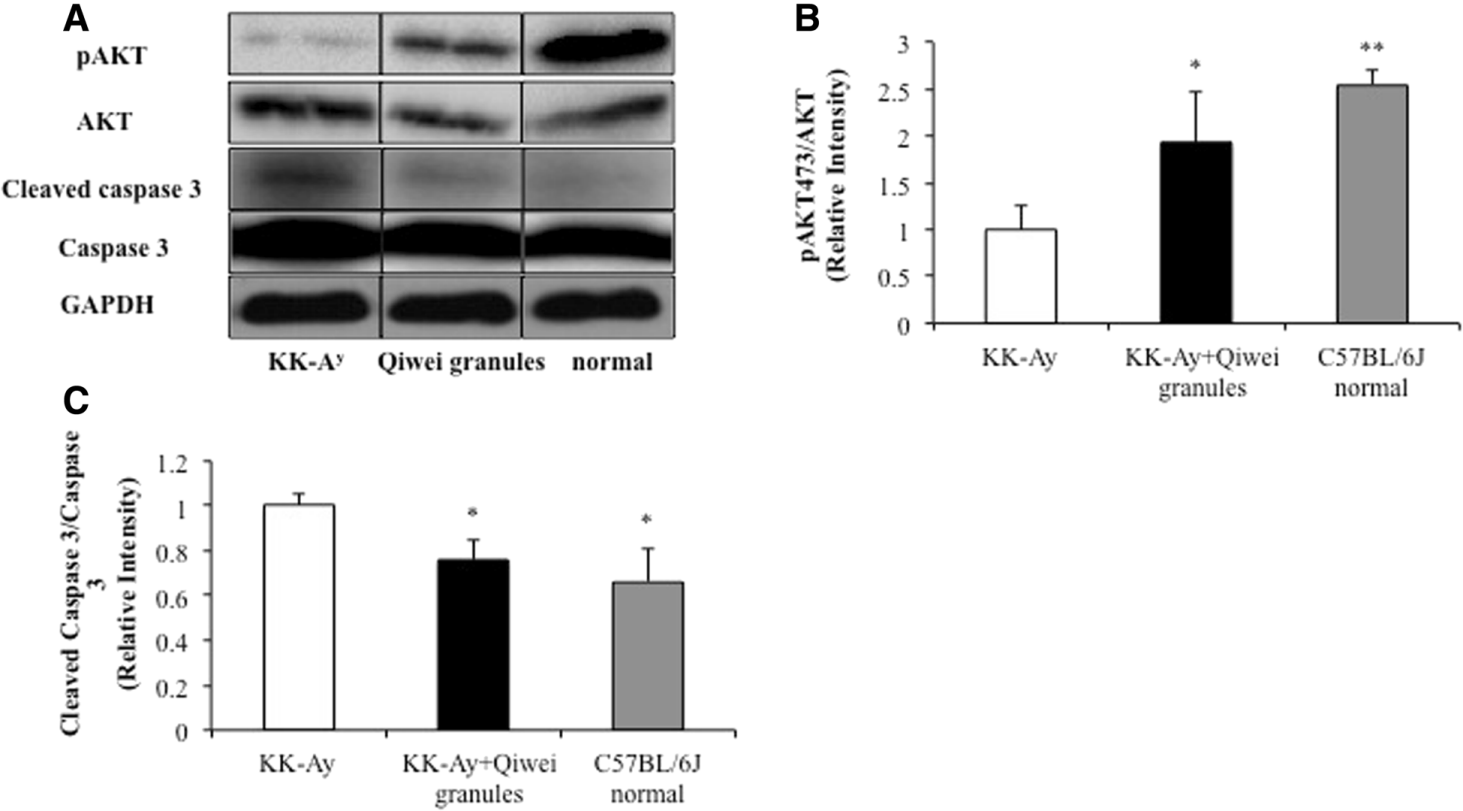
**Effect of Qiwei granules on phosphorylation of Akt and cleavage of caspase-3 signaling in the kidney of KK-A** ^**y**^ **mice. (A)** Protein expression levels of pAkt, Akt, cleaved caspase-3 and caspase-3 in renal tissues of three groups were analyzed by western blotting. Rate of Akt phosphorylation and caspase-3 activation were shown in **(B)** and **(C)**. Data were presented as mean ± SEM (n = 5) normalized to GAPDH protein expression. *P < 0.05; **P < 0.01 compared to KK-A^y^ vehicle group.

It has been shown that PI3K/AKT pathway related with SD molecules (SDs) is one key part of podocyte cell survival signaling to maintain podocyte functional integrity [28]. Our study found that Qiwei granules prevented the podocyte apoptosis and increase the expression of SDs, so we would investigate whether Qiwei granules regulated the phosphorylation of Akt and cleavage of caspase-3. As shown in Figure 7, the protein expression of pAkt in vehicle KK-A^y^ mice was significantly lower compared with normal mice, while the expression of total Akt was no difference between vehicle diabetic mice and normal mice. With Qiwei granules treatment, the expression of pAkt/Akt in KK-A^y^ mice was upregulated compared with vehicle KK-A^y^ mice. Further we examined the protein level of caspase-3 cleavage, the key downstream effector of apoptosis. The cleaved caspase-3 expression of vehicle diabetic mice was obviously higher than normal mice. Exposure to Qiwei granules resulted in decrease of cleaved caspase-3 expression in KK-A^y^ mice kindey. Thus, Qiwei granules improved the phosphorylation of Akt and inhibited cleavage of caspase-3.

## Discussion

Due to the constant increase in the incidence of type 2 diabetes mellitus, diabetic nephropathy has become the highly prevalent cause of chronic kidney disease worldwide. In China traditional Chinese medicines have been used for the diabetes mellitus and its complications for thousand of years. Chinese medicines have been attracted worldwide attention to become a new therapy for diabetic nephropathy. Qiwei granule, one of such traditional Chinese medicines, has been comprised of 6 Chinese herbs. In clinic study, Qiwei granule was shown to decrease blood glucose, improve hyperlipidemia, and reduce the proteinuria of DN patients [23]. The present study showed that Qiwei granules treatment significantly improved the metabolic parameters, alleviated the albuminuria, and protected renal structure to prevent progression of DN in KK-A^y^ mice. In addition, the mitigation of podocyte lesion with Qiwei granules treatment was further demonstrated, following by the increases of SD molecules expression and likely activating Akt signaling pathway.

The classical hallmarks of diabetic nephropathy include glomerular hypertrophy, expansion of mesangial matrix and thickness of GBM [29]. Recently mounting researches reveal that podoctye injury becomes a major contributor to the albuminuria in diabetic patients and animal models [3,4]. It relates to the importance of podocyte in maintaining the filtration barrier. However, the podocyte injuries including foot process effacement, detachment, hypertrophy, and apoptosis lead to the dyfunction of filtration barrier resulting in albuminuria [30,31]. In our study, the mesangial matrix expansion and GBM thickening were observed in KK-A^y^ mice. In addition, podocyte lesion demonstrated as foot process effacement and podocyte loss was found in KK-A^y^ mice. The treatment of Qiwei granules both reversed the glomerular and podocyte injuries.

Slit diaphragm molecules between podocytes composed of multiple protein complexes contribute to the glomerular filtration barrier. Derangement of SDs results in proteinuria. Nephrin is a major transmembrane protein located in the glomerular SD region. The altered expression of nephrin has been observed in the initiation of proteinuria and linked to the development of proteinuria in DN [29,32]. CD2AP, a transmembrane protein interacted with nephrin, preserves the functions of cytoskeleton and SD. Damage to CD2AP leads to disrupting podocyte cytoskeleton, and inducing massive proteinuria [9]. Basement membrane-podocyte junctional membrane proteins such as integrin α3β1 also play a vital role in preserving the normal structure and functioning of podocyte [13]. The downregulation of integrin α3β1 receptors detach podocytic processes from GBM and results in proteinuria [33]. Our finding suggested that expressions of nephrin, CD2AP, and integrin α3β1 was markedly decreased in diabetic KK-A^y^ mice compared with normal mice, whereas Qiwei granules treatment restored the SDs expressions. In combination, these results indicated that Qiwei granules treatment increased the expression levels of nephrin, CD2AP, and integrin α3β1 to protect from podocyte injury in diabetic KK-A^y^ mice.

Apoptosis, one of the considered causes of podocyte loss results in the development of albuminuria in diabetic nephropathy. Akt is a serine/threonine protein kinase that plays a critical role in cell survival by inhibiting the pro-apoptotic signals. Previous studies show that nephrin and CD2AP stimulate Akt signaling pathway and thereby reduce podocyte apoptosis to protect podocyte in glomerular [34,35]. Our results also showed that the expression of nephrin and CD2AP was decreased, while the phosphorylation of Akt was inhibited and induced the apoptosis in diabetic KK-A^y^ mice. Administration of Qiwei granules improved the phosphorylation of Akt and increased the expression of SDs contributing to preservation of podocyte. Caspase-3 is a critical executioner of apoptosis and operates as the key effector enzyme in cell death. Activation of caspase-3 plays an important role in apoptosis. In accordance with previous studies [36,37], our study showed that cleaved caspase-3 expression was increased in renal tissue of diabetic KK-A^y^ mice. Qiwei granules reduced the overexpression of cleaved caspase-3 in diabetic mice possibly leading to inhibit apoptosis. These results indicated that Qiwei granules maybe affect podocyte apoptosis depending on Akt pathway and caspase-3.

Previous studies are reported that high glucose induces podocyte apoptosis and reduction of SDs expressions contributing to podocyte injury [38]. In the present study, Qiwei granules decreased the blood glucose and protected the podocyte lesion to prevent the progress of DN. Qiwei granules consists of Astragalus membranaceus, Euonymus alatus, Rehmannia glutinosa, Prunella vulgaris, and Panax notoginseng that these herbs are shown to decreases the glucose, and protect diabetic nephropathy kidney [15-17,19,20]. In a word, Qiwei granules alleviates podocyte lesion maybe not only protecting podocyte directly, but also related with reducing blood glucose. This result requires further confirmation in the future.

## Conclusions

In conclusion, the Chinese formulation Qiwei granules protects podocyte in the kidney of diabetic KK-A^y^ mice. Qiwei granules treatment significantly improved the expression of SD molecules nephrin, CD2AP, integrin α3β1. Furthermore, it appears to activate Akt signaling pathway and inhibit the apoptosis in the kidney. It alleviated the podocyte lesion and glomerular pathologic changes, which resulted in the decrease of albuminuria and protection of renal function. Our study suggests that Qiwei granules ameliorates podocyte lesion to prevent progression of DN in KK-A^y^ mice.

## Abbreviations

BUN: Blood urine nitrogen
BW: Body weight
CD2AP: CD2 associated protein
DN: Diabetic nephropathy
ESRD: End-stage renal disease
FBG: Fasting blood glucose
GBM: Glomerular basement membrane
HE: Hematoxylin and eosin
PAS: Periodic acid-Schiff
Scr: Serum creatinine
SD: Slit diaphragm
TUNEL: TdT-mediated dUTP nick end labeling
WT-1: Wilms tumor −1

## References

[1] Whaley-Connell A, Sowers JR, McCullough PA, Roberts T, McFarlane SI, Chen SC (2009). Diabetes mellitus and CKD awareness: the Kidney Early Evaluation Program (KEEP) and National Health and Nutrition Examination Survey (NHANES). Am J Kidney Dis.

[2] Tervaert TW, Mooyaart AL, Amann K, Cohen AH, Cook HT, Drachenberg CB (2010). Pathologic classification of diabetic nephropathy. J Am Soc Nephrol.

[3] White KE, Bilous RW, Diabiopsies Study, G (2004). Structural alterations to the podocyte are related to proteinuria in type 2 diabetic patients. Nephrol Dial Transplant.

[4] Susztak K, Raff AC, Schiffer M, Bottinger EP (2006). Glucose-induced reactive oxygen species cause apoptosis of podocytes and podocyte depletion at the onset of diabetic nephropathy. Diabetes.

[5] Satchell SC, Braet F (2009). Glomerular endothelial cell fenestrations: an integral component of the glomerular filtration barrier. Am J Physiol Renal Physiol.

[6] Roselli S, Heidet L, Sich M, Henger A, Kretzler M, Gubler MC (2004). Early glomerular filtration defect and severe renal disease in podocin-deficient mice. Mol Cell Biol.

[7] Schwarz K, Simons M, Reiser J, Saleem MA, Faul C, Kriz W (2001). Podocin, a raft-associated component of the glomerular slit diaphragm, interacts with CD2AP and nephrin. J Clin Invest.

[8] Jim B, Ghanta M, Qipo A, Fan Y, Chuang PY, Cohen HW (2012). Dysregulated nephrin in diabetic nephropathy of type 2 diabetes: a cross sectional study. PLoS One.

[9] Toyoda M, Suzuki D, Umezono T, Uehara G, Maruyama M, Honma M (2004). Expression of human nephrin mRNA in diabetic nephropathy. Nephrol Dial Transplant.

[10] Benzing T (2004). Signaling at the slit diaphragm. J Am Soc Nephrol.

[11] Kagami S, Kondo S (2004). Beta1-integrins and glomerular injury. J Med Invest.

[12] Chen HC, Chen CA, Guh JY, Chang JM, Shin SJ, Lai YH (2000). Altering expression of alpha3beta1 integrin on podocytes of human and rats with diabetes. Life Sci.

[13] Kreidberg JA, Donovan MJ, Goldstein SL, Rennke H, Shepherd K, Jones RC (1996). Alpha 3 beta 1 integrin has a crucial role in kidney and lung organogenesis. Development.

[14] Fang J, Wei H, Sun Y, Zhang X, Liu W, Chang Q (2013). Regulation of podocalyxin expression in the kidney of streptozotocin-induced diabetic rats with Chinese herbs (Yishen capsule). BMC Complement Altern Med.

[15] Zhang J, Xie X, Li C, Fu P (2009). Systematic review of the renal protective effect of Astragalus membranaceus (root) on diabetic nephropathy in animal models. J Ethnopharmacol.

[16] Chang B, Jin C, Zhang W, Kong L, Yang JH, Lian FM (2012). Euonymus alatus in the treatment of diabetic nephropathy in rats. Am J Chin Med.

[17] Shieh JP, Cheng KC, Chung HH, Kerh YF, Yeh CH, Cheng JT (2011). Plasma glucose lowering mechanisms of catalpol, an active principle from roots of Rehmannia glutinosa, in streptozotocin-induced diabetic rats. J Agric Food Chem.

[18] Zheng J, He J, Ji B, Li Y, Zhang X (2007). Antihyperglycemic activity of Prunella vulgaris L. in streptozotocin-induced diabetic mice. Asia Pac J Clin Nutr.

[19] Valentova K, Truong NT, Moncion A, de Waziers I, Ulrichova J (2007). Induction of glucokinase mRNA by dietary phenolic compounds in rat liver cells in vitro. J Agric Food Chem.

[20] Hwang SM, Kim JS, Lee YJ, Yoon JJ, Lee SM, Kang DG (2012). Anti-diabetic atherosclerosis effect of Prunella vulgaris in db/db mice with type 2 diabetes. Am J Chin Med.

[21] Guang-Rong Z, Zhi-Jun X, Ting-Xiang Y (2006). Antioxidant activities of Salvia miltiorrhiza and Panax notoginseng. Food Chem.

[22] Peng SL, Guo ZA (2010). Effect of total saponins of Panax notoginseng on urinary albumin in patients with chronic renal failure. Chinese Critical Care Medicine.

[23] Jinxi Z, Xin M, Shidong W (2004). Therapeutic Observations on Renal Insufficiency of Diabetic Nephropathy in Its Compensatory Phase Treated by Zhixiao Tongmai Ning Granule: A Report of 33 Cases. Henan Traditional Chinese Medicine.

[24] Minzhou L, Yanbin G, Mingfei M (2013). The effect of Qiwei granules on TGF-β1/Smads signaling pathway in KK-Ay mice. J Tradit Chin Med.

[25] Iwatsuka H, Shino A, Suzuoki Z (1970). General survey of diabetic features of yellow KK mice. Endocrinol Jpn.

[26] Castle CK, Colca JR, Melchior GW (1993). Lipoprotein profile characterization of the KKA(y) mouse, a rodent model of type II diabetes, before and after treatment with the insulin-sensitizing agent pioglitazone. Arterioscler Thromb.

[27] Okazaki M, Saito Y, Udaka Y, Maruyama M, Murakami H, Ota S (2002). Diabetic nephropathy in KK and KK-Ay mice. Exp Anim.

[28] Xavier S, Niranjan T, Krick S, Zhang T, Ju W, Shaw AS (2009). TbetaRI independently activates Smad- and CD2AP-dependent pathways in podocytes. J Am Soc Nephrol.

[29] Jefferson JA, Shankland SJ, Pichler RH (2008). Proteinuria in diabetic kidney disease: a mechanistic viewpoint. Kidney Int.

[30] Satchell SC, Tooke JE (2008). What is the mechanism of microalbuminuria in diabetes: a role for the glomerular endothelium?. Diabetologia.

[31] Wolf G, Chen S, Ziyadeh FN (2005). From the periphery of the glomerular capillary wall toward the center of disease: podocyte injury comes of age in diabetic nephropathy. Diabetes.

[32] Aaltonen P, Luimula P, Astrom E, Palmen T, Gronholm T, Palojoki E (2001). Changes in the expression of nephrin gene and protein in experimental diabetic nephropathy. Lab Invest.

[33] Pozzi A, Jarad G, Moeckel GW, Coffa S, Zhang X, Gewin L (2008). Beta1 integrin expression by podocytes is required to maintain glomerular structural integrity. Dev Biol.

[34] Huber TB, Hartleben B, Kim J, Schmidts M, Keil A, Egger L (2003). Nephrin and CD2AP associate with phosphoinositide 3-OH kinase and stimulate AKT-dependent signaling. Mol Cell Biol.

[35] Bridgewater DJ, Ho J, Sauro V, Matsell DG (2005). Insulin-like growth factors inhibit podocyte apoptosis through the PI3 kinase pathway. Kidney Int.

[36] Tuncdemir M, Ozturk M (2011). The effects of angiotensin-II receptor blockers on podocyte damage and glomerular apoptosis in a rat model of experimental streptozotocin-induced diabetic nephropathy. Acta Histochem.

[37] Gui D, Guo Y, Wang F, Liu W, Chen J, Chen Y (2012). Astragaloside IV, a novel antioxidant, prevents glucose-induced podocyte apoptosis in vitro and in vivo. PLoS One.

[38] Li C, Siragy H (2014). High glucose induces podocyte injury via enhanced (pro)renin Receptor-Wnt-β-Catenin-Snail signaling. PLoS One.

